# Utility of Whole-Body Magnetic Resonance Imaging Surveillance in Children and Adults With Cancer Predisposition Syndromes: A Retrospective Study

**DOI:** 10.1200/PO-24-00642

**Published:** 2025-03-26

**Authors:** Frances Victoria Fajardo Que, Nur Diana Binte Ishak, Shao-Tzu Li, Jeanette Yuen, Tarryn Shaw, Hui Xuan Goh, Zewen Zhang, Jianbang Chiang, Si Yong Yeo, Lee Lian Chew, Choon Hua Thng, Joanne Ngeow

**Affiliations:** ^1^ Cancer Genetics Service, Division of Medical Oncology, National Cancer Centre Singapore, Singapore; ^2^ Lee Kong Chian School of Medicine, Nanyang Technological University, Singapore; ^3^ Division of Oncologic Imaging, National Cancer Centre Singapore, Singapore

## Abstract

**PURPOSE:**

Surveillance improves patient outcomes by diagnosing cancer at an early and curable stage for individuals with cancer predisposition syndromes (CPS). Whole-body magnetic resonance imaging (WB-MRI) provides head-to-thigh imaging in one sitting without radiation exposure and is recommended for individuals with increased risk of multisite tumors or cancers. In this study, we evaluated the diagnostic performance of WB-MRI as a screening tool for Li-Fraumeni syndrome (LFS), constitutional mismatch repair deficiency syndrome, hereditary paraganglioma-pheochromocytoma syndrome (HPGL-PCC), and other CPS.

**METHODS:**

A retrospective review of patients with CPS seen at the Cancer Genetics Service, National Cancer Center Singapore was conducted. Patients who underwent WB-MRI screening from 2014 to 2024 were identified to determine the sensitivity and specificity of WB-MRI in early cancer detection.

**RESULTS:**

Of the 103 patients with CPS recommended for WB-MRI surveillance, 59 underwent the procedure (57% uptake rate). Among them, 34 (57%) were female and the median age was 32 years (range, 1-74 years). The CPS distributions included 22 (37%) with LFS, 14 (24%) with neurofibromatosis type 1, eight (14%) with Von Hippel Lindau syndrome, and eight (14%) with HPGL-PCC. WB-MRI screening led to a diagnosis of cancer in seven (12%) patients (renal cell carcinoma, prostate adenocarcinoma, osteosarcoma, neuroendocrine tumor); of these, four (57%) received curative treatment. Twelve (20%) patients required additional investigations, with eight (14%) having benign findings. The sensitivity and specificity of WB-MRI in our cohort were 64% and 92%, respectively, with a false-positive rate (FPR) of 8% and a false-negative rate of 36%.

**CONCLUSION:**

WB-MRI is an effective screening tool in patients with specific CPS, demonstrating a high specificity and low FPR.

## INTRODUCTION

Individuals with cancer predisposition syndromes (CPS) have significantly increased cancer incidence and mortality. It is challenging to develop effective protocols for surveillance in these high-risk patients, which is complicated by multisite risk, incomplete penetrance, and variable expressivity. Still, surveillance holds great potential to improve patient outcomes by diagnosing cancer at an early and curable stage, and currently is the least invasive option these individuals have in managing their cancer risk.^1^

**CONTEXT**

**Key Objective**
What is the diagnostic performance of whole-body magnetic resonance imaging (WB-MRI) as a screening tool for Li-Fraumeni syndrome, constitutional mismatch repair deficiency syndrome, hereditary paraganglioma-pheochromocytoma syndrome, and other cancer predisposition syndromes (CPS)?
**Knowledge Generated**
In this retrospective study of 103 patients with CPS recommended for WB-MRI surveillance, 59 underwent the procedure (57% uptake rate). WB-MRI screening led to a diagnosis of cancer in seven (12%) patients (renal cell carcinoma, prostate adenocarcinoma, osteosarcoma, neuroendocrine tumor); of these, four (57%) received curative treatment. The sensitivity and specificity of WB-MRI in our cohort were 64% and 92%, respectively, with a false-positive rate (FPR) of 8% and a false-negative rate of 36%.
**Relevance**
WB-MRI is an effective screening tool in patients with specific CPS, demonstrating a high specificity and low FPR, leading to early cancer detection and treatment.

Whole-body magnetic resonance imaging (WB-MRI) has emerged as a recommended imaging procedure for surveillance of patients with certain cancer predisposition conditions, as it provides whole-body imaging in a single sitting without exposure to ionizing radiation, enabling efficient assessment of different organs for multifocal diseases.^2,3^

There are three CPS that have established guidelines on the use of WB-MRI—Li-Fraumeni syndrome (LFS), constitutional mismatch repair deficiency syndrome (CMMRD), and hereditary paraganglioma-pheochromocytoma syndrome (HPGL-PCC).^3^

There are other CPS where WB-MRI might be beneficial. These include neurofibromatosis type 1 (NF1), Von Hippel Lindau (VHL), multiple endocrine neoplasia type 1 (MEN1), Carney complex (CC), and Fanconi anemia (FA).^4-8^

The Cancer Genetics Service (CGS), National Cancer Center Singapore (NCCS), manages patients with these genetic conditions and bases its clinical practice and management on recommended international guidelines, as well as on the guidelines developed by the Cancer Service Line Development Workgroup for Cancer Genetics of NCCS, which is a consensus by a team of multidisciplinary specialists in Singapore (2021, Version 1; Table 1).

**TABLE 1.** Guidelines on the Use of WB-MRI for Patients With CPSs

CPS	CGS, NCCS Practice	International Guidelines/Recommendations	
	Imaging Recommendation	Imaging Recommendation	Reference
LFS	Annual WB-MRI +brain MRI from birth +breast MRI in women from age 20 years	Annual WB-MRI +brain MRI with contrast (+breast MRI in women) for pediatric and adult patients	NCCN^9^ and AACR^10^
CMMRD	6-12 monthly brain MRI from age 2 years	Annual WB-MRI from age 6 years	Care for CMMRD Consortium & International Biallelic Mismatch Repair Deficiency Consortium, EviQ ^11,12^
HPGL-PCC	WB-MRI every 5 years from age 18 years for *SDHA*, Every 2 years from age 10 years for *SDHB* Every 3-5 years from age 18 years for *SDHC* and *SDHAF2* Every 3-5 years from age 10 years for *SDHD*	Biennial WB-MRI	AACR^13^
NF1	Baseline WB-MRI should be considered between ages 16 and 20 years to assess internal tumor burden to determine adult follow-up regimen	Suggested to introduce WB-MRI for the detection of MPNSTs	NCCN^14^
VHL syndrome	MRI of the brain, spine, and abdomen every 2 years	MRI of the brain, spine, and abdomen every 2 years	VHL Alliance ^5^
MEN1	Age 10 years: Baseline high-resolution pituitary MRI (combined with adrenal and/or chest) Age 20 years: 1-2 yearly WB-MRI 4 yearly high-resolution pituitary MRI (combined with adrenal and/or chest imaging)	MRI of the brain, every 3-5 years, beginning between ages 5 and10 years, or at any time the results of the tests for serum prolactin or insulin-like growth factor are abnormal MRI or CT scan of the chest and abdomen, every 2-4 years, beginning at age 20 years or when the serum gastrin, PP, or VIP is noted to be abnormal	ASCO; EviQ^6,15^
CC (*PRKAR1A*)	WB-MRI, when clinically indicated	Annual or biannual echocardiogram; annual testicular ultrasound and baseline thyroid and transabdominal ultrasound of the ovaries with repeat as necessary Further evaluation with adrenal CT scan and MRI (pituitary, brain, spine, chest, abdomen, retroperitoneum, pelvis) as needed	Stratakis et al^7^
FA	WB-MRI, when clinically indicated	Close monitoring (ie, MRI of pituitary gland, MRI of breast)	Fanconi Anemia Research Fund^8^

Abbreviations: AACR, American Association for Cancer Research; CC, Carney complex; CGS, Cancer Genetics Service; CMMRD, constitutional mismatch repair deficiency syndrome; CPS, cancer predisposition syndromes; FA, Fanconi anemia; HPGL-PCC, hereditary paraganglioma-pheochromocytoma syndrome; LFS, Li-Fraumeni syndrome; MEN1, multiple endocrine neoplasia type 1; NCCN, National Comprehensive Cancer Network; NCCS, National Cancer Center Singapore; NF1, neurofibromatosis type 1; PP, pancreatic polypeptide; VHL, Von Hippel Lindau; VIP, vasoactive intestinal peptide; WB-MRI, whole-body magnetic resonance imaging.

There is currently limited information in both oncology and radiology literature on the use of WB-MRI as an accurate and effective screening modality for patients with CPS. In this study, we aim to describe our experience with offering WB-MRI as a screening modality to individuals with CPS to determine its diagnostic performance as a screening tool.

## METHODS

### Patient Selection

We identified all patients with LFS, CMMRD, HPGL-PCC, NF1, VHL, MEN1, CC, and FA who were seen at the CGS, NCCS, and underwent WB-MRI from 2014 to 2024. These are the CPS for which WB-MRI is recommended according to local and international guidelines. Eligible patients include all children and adults, had a molecularly confirmed diagnosis, had complete imaging and medical records, and had undergone at least one WB-MRI examination for tumor surveillance.

### MRI Imaging

All WB-MRI examinations were performed with a 1.5-Tesla MRI unit (Siemens Aera, Erlangen, Germany). Anatomical coverage was from the calvarial vertex to the mid-thighs.

Core sequences included axial T2 HASTE with fat suppression, axial T1-weighted gradient echo, coronal T2 TIRM, and coronal T1-weighted turbo spin echo for morphologic imaging. Diffusion-weighted imaging (DWI) sequences were acquired in the axial plane with *b* values of 50 and 800 s/mm^2^, providing functional information. DWI was omitted in some conditions such as VHL and NF1 to keep examination times as short as possible. Images were obtained in 5- to 6-mm slice thickness. The MRI study was performed in four parts anatomically with overlap: head and neck, chest, abdomen, and thighs. Commercially available stitching software on our Siemens unit was used to stitch the images together into a single composed sequence. Additional sequences were then tailored according to the specific risk profile of the underlying CPS. For example, axial whole-body postcontrast T1-weighted sequences with fat suppression were added in patients with LFS, NF1, VHL, and MEN1 to increase sensitivity for lesion detection. In conditions where there is increased risk of CNS tumors, dedicated sequences of the brain (axial T2 FLAIR, T1-weighted, and postcontrast sequences) were performed. When there is increased risk of intra-abdominal neoplasms, multiphasic postcontrast VIBE sequences of the abdomen were added to the protocol. The total scan time ranged from 35 to 50 minutes.

### Medical Record Review

Patient records were reviewed retrospectively; demographic information, germline genetic test results, personal history of malignancy, family history of malignancy, and any treatment the patient received were noted. In our cohort, patients without a previous cancer diagnosis underwent WB-MRI primarily for screening, whereas those with a history of cancer underwent WB-MRI for surveillance in accordance with international guidelines. For patients undergoing surveillance, WB-MRI was used to monitor for potential recurrences or the development of additional cancers associated with their CPS. For patients who underwent more than one WB-MRI examination, the interval between the studies was reviewed. Abnormal findings on WB-MRI were correlated with the patient's medical records for relevant histopathology, laboratory data, and radiologic follow-up studies.

### Statistical Analysis

Each WB-MRI finding was classified as a true positive, true negative, false positive, or false negative. A WB-MRI finding was considered a true positive if a malignancy was subsequently diagnosed and a true negative if no abnormality was detected and the patient remained well. A false positive was defined as a suspicious lesion in a patient who did not have clinical, pathologic, or further imaging findings to confirm malignancy, whereas it was classified as a false negative when a lesion suggestive of malignancy was not detected, but the patient was subsequently diagnosed with a tumor related to a CPS. On the basis of these parameters, the sensitivity, specificity, positive predictive value (PPV), and negative predictive value (NPV) were calculated for WB-MRI as a screening tool for the overall study cohort, as well as for each CPS.^1^

## RESULTS

Overall, there were 103 patients with CPS who were recommended WB-MRI as a screening modality, of which 59 patients underwent WB-MRI (57% uptake), receiving a total of 126 WB-MRIs collectively (Table 2). Patients' median age was 32 years (range, 1-74). There were more females (57%) than males (43%), mostly Chinese (83%), and the majority of patients had a personal history (75%) and/or family history of cancer (80%). Patients had an average of two WB-MRI examinations done over an interval ranging from 3 months to 3 years (Table 3).

**TABLE 2.** Distribution of CPS Recommended and Underwent WB-MRI

CPS	Total Recommended WB-MRI (N = 103)	Underwent WB-MRI (N = 59)	Recommended Patients Who Underwent WB-MRI, %	Reasons for Declining WB-MRI
LFS	33	22	66.7	Cost, claustrophobia, personal choice, undergoing active treatment
NF1	27	14	51.9	Cost, anxiety, lack of perceived benefit
VHL syndrome	12	8	66.7	Undergoing active treatment, personal choice
Hereditary paraganglioma-pheochromocytoma	21	8	38.1	Cost, lost to follow-up, undergoing active treatment, lack of perceived benefit, personal choice
MEN1	7	3	42.9	Cost, lack of perceived benefit
FA	1	1	100	NA
CC	2	0	0	Limited data
Total	103	59	57.3	Mixed reasons as indicated above

Abbreviations: CC, Carney complex; CPS, cancer predisposition syndromes; FA, Fanconi anemia; LFS, Li-Fraumeni syndrome; MEN1, multiple endocrine neoplasia type 1; NA, not applicable; NF1, neurofibromatosis type 1; VHL, Von Hippel Lindau; WB-MRI, whole-body magnetic resonance imaging.

**TABLE 3.** Baseline Characteristics of Patients Who Underwent Whole-Body Magnetic Resonance Imaging (N = 59)

Demographic Categories	No. (%)
Age, years	
<20	8
21-40	31
41-60	18
>60	2
Sex	
Male	25 (43)
Female	34 (57)
Race	
Chinese	49 (83)
Indian	6 (10)
Malay	3 (5)
Others	1 (2)
Personal history of cancer	
Yes	44 (75)
No	15 (25)
Family history of cancer	
Yes	47 (80)
No	12 (20)
CPS	
LFS^a^	22 (37)
NF1^a^	14 (24)
LFS and NF1	1 (2)
VHL syndrome	8 (14)
Hereditary paraganglioma-pheochromocytoma syndrome	8 (14)
MEN1	3 (5)
FA	1 (2)
CC	2 (3)
Number of examinations done per patient	
1	26 (44)
>1	33 (56)

Abbreviations: CC, Carney complex; CPS, cancer predisposition syndromes; FA, Fanconi anemia; LFS, Li-Fraumeni syndrome; MEN1, multiple endocrine neoplasia type 1; NF1, neurofibromatosis type 1; VHL, Von Hippel Lindau.

^a^ A patient is counted twice because of having both LFS and neurofibromatosis type 1.

Among the 59 patients who underwent WB-MRI, 15 individuals were found to have abnormal findings, whereas 40 demonstrated unremarkable results. Within the subset of 15 patients displaying aberrant outcomes, seven were diagnosed with cancers through WB-MRI (three patients) or in conjunction with additional imaging (four patients). Conversely, among those patients reported to have normal outcomes, four individuals were subsequently diagnosed with interval cancers (defined as an invasive cancer diagnosed following a negative screening result and before the next recommended examination is due)^16^ through other screening modalities.

For the 11 patients identified with cancers, the type of cancers were prostate adenocarcinoma, osteosarcoma, breast ductal carcinoma, leukemia, renal cell carcinoma, pancreatic neuroendocrine tumor, and tongue squamous cell carcinoma. Of the 11 cancers detected, three were identified directly by WB-MRI, four were detected through additional imaging prompted by WB-MRI findings, and the remaining four were discovered through interval screening using other imaging modalities, prompted from clinical suspicion (Table 4).

**TABLE 4.** Outcomes of Patients' Diagnosis From Various Screening Modalities

Screening Modality	Total Patients (N = 59)	Method of Confirmation	%
WB-MRI only	38		64
No tumor/cancer detected	35		59
Tumor/cancer detected	3		5
Types of tumor/cancer detected			
Clear cell papillary renal cell carcinoma	1	Pathology	
Prostate adenocarcinoma	1	Pathology	
Osteosarcoma	1	Pathology	
WB-MRI + additional imaging^a^	12		20
No tumor/cancer detected	8		14
Tumor/cancer detected	4		7
Types of tumor/cancer detected			
Neuroendocrine tumor	2	Pathology	
Osteosarcoma	1	Pathology	
Suspicious liver and lung lesions	1	Imaging alone (patient declined biopsy)	
Other interval screening	4		7
Breast MRI	2		
Tumor/cancer detected	2		
Types of tumor/cancer detected			
Breast cancer	2	Pathology	
History and physical examination			
Tumor/cancer detected	1		
Types of tumor/cancer detected			
Squamous cell carcinoma, tongue	1	Pathology	
Full blood count			
Tumor/cancer detected	1		
Types of tumor/cancer detected			
Leukemia	1	Laboratory evaluation	

Abbreviations: CT, computed tomography; MRI, magnetic resonance imaging; WB-MRI, whole-body magnetic resonance imaging.

^a^ Additional imaging includes modalities such as CT scan of specific organs, bone scan, or whole-body positron emission tomography CT scan.

On the basis of these findings, the diagnostic performance of WB-MRI among our cohort of patients with CPS was as follows: sensitivity, 64%; specificity, 92%; PPV, 64%; and NPV, 92%. The calculated false-positive rate (FPR) and false-negative rate (FNR) were 8% and 36%, respectively (Table 5).

**TABLE 5.** Outcomes of 59 Patients Who Underwent WB-MRI

WB-MRI	Cancer	No Cancer
Abnormal finding	7^a^	4
Negative	4	44

NOTE. Sensitivity: 64%; specificity: 92%; PPV: 64%; NPV: 92%; FPR: 8%; FNR: 36% (limitations: breast cancer; leukemia; tongue SCC).

Abbreviations: FNR, false-negative rate; FPR, false-positive rate; NPV, negative predictive value; PPV, positive predictive value; SCC, squamous cell carcinoma; WB-MRI, whole-body magnetic resonance imaging.

^a^ Four cancers treated with curative intent.

### LFS

Among the 22 patients with LFS who underwent WB-MRI, seven were diagnosed with cancer—two were detected by WB-MRI alone, and one had cancer treated with a curative intent (Table 6).

**TABLE 6.** Patients with Li-Fraumeni Syndrome Who Underwent WB-MRI Screening and Were Diagnosed With Cancer

No.	Age, Years	Race	Gender	History	Fhx of Ca	No. of Annual WB-MRI	Further Imaging	Additional Procedure	Cancer Diagnosis	Outcome
1	40	China	Male	Sarcoma	Yes	3	Nil	Biopsy and surgery	Prostate adenocarcinoma	Curative treatment
2	8	China	Male	Rhabdomyosarcoma	Yes	4	Nil	Surgery	Osteosarcoma (right clavicle)	Deceased
3	13	China	Male	Osteosarcoma	Yes	3	CT chest	Biopsy (not done, patient deceased)		Deceased
4	48	India	Female	Osteosarcoma, rectal adenocarcinoma	Yes	3	Breast MRI	Surgery	Breast cancer	Curative treatment
							PET CT scan	Biopsy (not done—patient refused)	Observing liver and lung lesions (likely recurrence, imaging-based conclusion)	Palliative intent
5	43	China	Female	Nil cancer	Yes	3	MMG/Breast UTZ (2018—unremarkable)	2019 Breast MRI and biopsy, left breast	Left breast infiltrative ductal carcinoma, ER+ PR– HER2+, stage IV (metastatic to bone)	Palliative intent
6	14	China	Male	Medulloblastoma	Yes	1	Nil	No	Leukemia	Deceased

Abbreviations: CT, computed tomography; ER, estrogen receptor; HER2, human epidermal growth factor receptor 2; PET, positron emission tomography; PR, progesterone receptor; WB-MRI, whole-body magnetic resonance imaging.

Patient 1 is a 40-year-old Chinese male with LFS, diagnosed with sarcoma in 2015, who underwent WB-MRI in 2018 which was unremarkable. In 2019, his WB-MRI showed a 1.2-cm lesion in the prostate gland, suspicious for tumor. He underwent a biopsy, and subsequently robotic prostatectomy and lymph node dissection for prostate adenocarcinoma, Gleason 4 + 3 = 7, stage IIIB (pT3N0M0). His WB-MRI in 2020 was unremarkable.

Patient 2 presented to CGS at age 1 years with left forearm rhabdomyosarcoma in 2009 and underwent excision, radiotherapy, and chemotherapy. He did interval follow-ups with MRI which were unremarkable, until 2016 at age 8 years when he was diagnosed with left forearm osteosarcoma and subsequently underwent chemotherapy and left arm amputation. His WB-MRI done in 2018 was normal. However, WB-MRI done in 2019 at age 11 years showed a right clavicle mass, leading to a diagnosis of osteosarcoma with lung and bone metastases. He was given systemic treatment followed by multiple resection surgeries and metastasectomies but responded poorly and died in November 2020 at age 12 years.

### VHL

Among the eight patients with VHL who underwent WB-MRI, one was diagnosed with cancer (renal cell carcinoma; cancer detection rate of 13%) and received treatment with curative intent.

This patient is a 32-year-old Chinese female with VHL but no previous history of cancer who underwent WB-MRI in 2018 which showed a 1-cm lesion in the left kidney. She underwent left partial nephrectomy and was diagnosed with clear cell papillary renal cell carcinoma, stage I. WB-MRI done in 2020 was unremarkable.

### MEN1

Three patients with MEN1 had screening WB-MRI. One was diagnosed with cancer (one neuroendocrine tumor) and was treated with curative intent.

This is a 33-year-old Chinese male with MEN1, previously diagnosed with insulinoma, who underwent WB-MRI and was found to have a pancreatic lesion, for which he underwent computed tomography (CT) scan of the pancreas and surgery to work up and remove a well-differentiated neuroendocrine tumor, stage III (T1(2)N1M0).

### FA

We had one patient with FA, a 28-year-old Chinese male with a previous history of lymphoma, who had a total of three annual WB-MRIs. However, before the next recommended examination was due, he presented with a white plaque on his tongue with intermittent bleeding which worsened over a month. MRI of the neck showed faint superficial signal abnormality in the left hemi tongue. He subsequently underwent left partial glossectomy for a stage I (pT1pN0cM0) tongue squamous cell carcinoma.

### Other CPS

None of the patients with HPGL-PCC, NF1, and CC who had WB-MRI screening were diagnosed with cancer. All are alive.

## DISCUSSION

We initiated this single-center investigation to assess our experience with WB-MRI and evaluate its diagnostic performance as a cancer screening tool in high-risk patients with CPS. We identified 15 abnormalities which warranted further action such as additional imaging, biopsy, or surgery, resulting in a 12% overall cancer detection rate, calculated as seven cancers detected out of 59 patients with CPS who underwent WB-MRI. On the basis of our findings, the sensitivity and specificity of WB-MRI were 64% and 92%, respectively. NPV was high at 92%, indicating that a normal WB-MRI is reassuring for patients with elevated risk of cancer due to an underlying CPS.

The true-positive rate of 64% emphasizes the patients who derive the most benefit from WB-MRI screening—those patients with LFS, MEN1, and VHL, whose cancers were found at an early, nonmetastatic stage and were treated with curative intent via surgery and systemic therapy.

The FNR of 36% is low; however, there are limitations of using WB-MRI as a screening method, as breast cancer, leukemia, and tongue squamous cell carcinoma are best diagnosed using other modalities, and any imaging procedure should be done as an adjunct to a thorough clinical history and physical examination. Despite this limitation, our patients still derived benefit from close monitoring and follow-up. This is demonstrated by our patient diagnosed with tongue squamous cell carcinoma, whose cancer was detected on subsequent follow-up at an early stage and treated with curative intent.

Given that WB-MRI is highly sensitive, it can pick up nonspecific abnormalities; hence, caution of overscreening should be kept in mind, as there is a possible impact on quality of life and patient anxiety. One of our patients who had WB-MRI screening was found to have a pancreatic nodule of which EUS and fine-needle aspiration biopsy were nondiagnostic. This case highlights the risk of having false-positive results with MRI imaging, with possible repercussions on quality of life and patient anxiety; thus, there is a need to further tailor surveillance protocols on the basis of the local context and specific cancer risks. In a study by Schmidt et al, it was found that although WB-MRI can generate distress and anxiety in the short term, it is generally well accepted in the long term, with quality of life and subjective stress levels comparable with those of other pre-existing cancer screening programs.^3,17,18^ Most patients in our cohort (35 of 59, 59%) had findings of no significant clinical impact.

In previous studies done on patients with LFS who underwent WB-MRI, cancer detection rates were approximately 7%, and false-positive rates ranged from 29.6% to 42.5%, with most screen-detected new cancers treated with curative intent.^19,20^ We had a higher cancer detection rate (29%) compared with other studies on WB-MRI screening for patients with LFS.

In a previous study on patients with VHL who underwent WB-MRI, the cancer detection rate was 5%, and screen-detected new cancers were treated with curative intent.^4^ We had a higher cancer detection rate (13%). Similarly, screen-detected new cancers were treated with curative intent.

False-positive cancer screening results are common and may affect patients' willingness to continue cancer screening in the future.^21^ With a high specificity and a low FPR, our results show that WB-MRI performs well for screening because of low false-positive errors, which decreases unnecessary psychological consequences and lessens the need for additional invasive and costly follow-up diagnostic or therapeutic procedures. This may be explained by the use of different MRI protocols, which leads to different diagnostic performance of WB-MRI among different institutions. Reporting and interpretation of WB-MRI findings may also vary by experience of the radiologist and interpreter skill.^21-23^

Studies such as ours highlight the benefits of genetic testing and radiologic screening for early detection of occult malignancies in patients with CPS. Knowledge of specific cancer predispositions and increased multifocal cancer risks can educate patients and clinicians on the need for WB-MRI and improve long-term outcomes by maximizing survival and minimizing morbidity from cancers and the treatment.

Finally, we had at least 103 patients who were advised to do WB-MRI surveillance, but only 59 (57%) underwent the procedure, translating to underutilization of this modality. Several factors may explain this, including socioeconomic status, cost, lack of reimbursements, patient burnout/fatigue, claustrophobia, anxiety, undergoing active treatment, lack of perceived benefit, personal choice, being lost to follow-up, and poor adherence to screening, resulting in noncompliance. Previous studies cite high costs of clinical genetic testing as the main barrier to patient's willingness to test, but provision of government subsidy leads to a considerable increase in genetic testing uptake rate and reduces the total cost of cancer management.^24^ The high cost of WB-MRI surveillance also adds on to the high costs of clinical genetic testing.

This study is limited by a small sample size, with data obtained over a short period of time, which may underestimate the benefits of WB-MRI. However, CPS are rare, and there is a paucity of data on the use of WB-MRI as a cancer screening method. Despite these limitations, this study provides important data with a range of clinical implications. With larger populations of patients with CPS followed up over a longer period, more data will emerge which may better define the timing and frequency of WB-MRI screening and determine the benefits and risks of this procedure. Estimates of the cost-effectiveness of WB-MRI also lie beyond the scope of this study but will be important information for implementation into clinical practice.

In conclusion, WB-MRI is an effective screening tool in patients with specific CPS, demonstrating a high specificity and low FPR, leading to early cancer detection and treatment. Genetic testing to identify patients at increased risk of cancer, together with radiologic screening for early detection of occult malignancies in patients with specific CPS, holds great potential to improve long-term outcomes by maximizing survival and minimizing morbidity from cancers and their treatment.

## Data Availability

The data that support the findings of this study are available on request from the corresponding author J.N. The data are not publicly available due to them containing information that could compromise research participant privacy/consent.
